# Supplementary material for: A generalizable Cas9/sgRNA prediction model using machine transfer learning with small high-quality datasets
Source: Nat Commun. 2023 Sep 7;14:5514. doi: 10.1038/s41467-023-41143-7 (PMC10485023; doi:10.1038/s41467-023-41143-7)
Supplement: Supplementary file 1 — Supplementary Information [file 41467_2023_41143_MOESM1_ESM.pdf]

Supplementary Figures for:
A generalizable Cas9/sgRNA prediction model using machine transfer learning with small high-quality datasets

Dalton T. Ham[1*], Tyler S. Browne[1*], Pooja N. Banglorewala[1], Tyler Wilson[2], Richard Michael[2], Gregory B. Gloor[1+], and David R. Edgell[1+]

[1]Department of Biochemistry, Schulich School of Medicine and Dentistry, London, ON, N6A5C1, Canada
[2]Tesseraqt Optimization Inc, Toronto, ON, Canada
[*]These authors contributed equally to this work
[+]correspondence to: dedgell@uwo.ca, ggloor@uwo.ca

1. Figure S1. Eukaryotic model testing on the TevSpCas9 and SpCas9 datasets.
2. Figure S2. Plasmid maps.
3. Figure S3. TevSpCas9 schematic.
4. Figure S4. PAM preference for TevSpCas9 and SpCas9.
5. Figure S5. sgRNAs drop out of cloning pool.
6. Figure S6. Off-target sgRNA predictions and respective activities in pooled assay.
7. Figure S7. Growth curves for sgRNAs tested individually.
8. Figure S8. Activity scoring across datasets.
9. Table S1. Area under the curve calculations from Growthcurver
10. Table S2. Off-targets in *E. coli* genome sgRNAs toxic in growth curves
11. Table S3. List of primers used in this study.

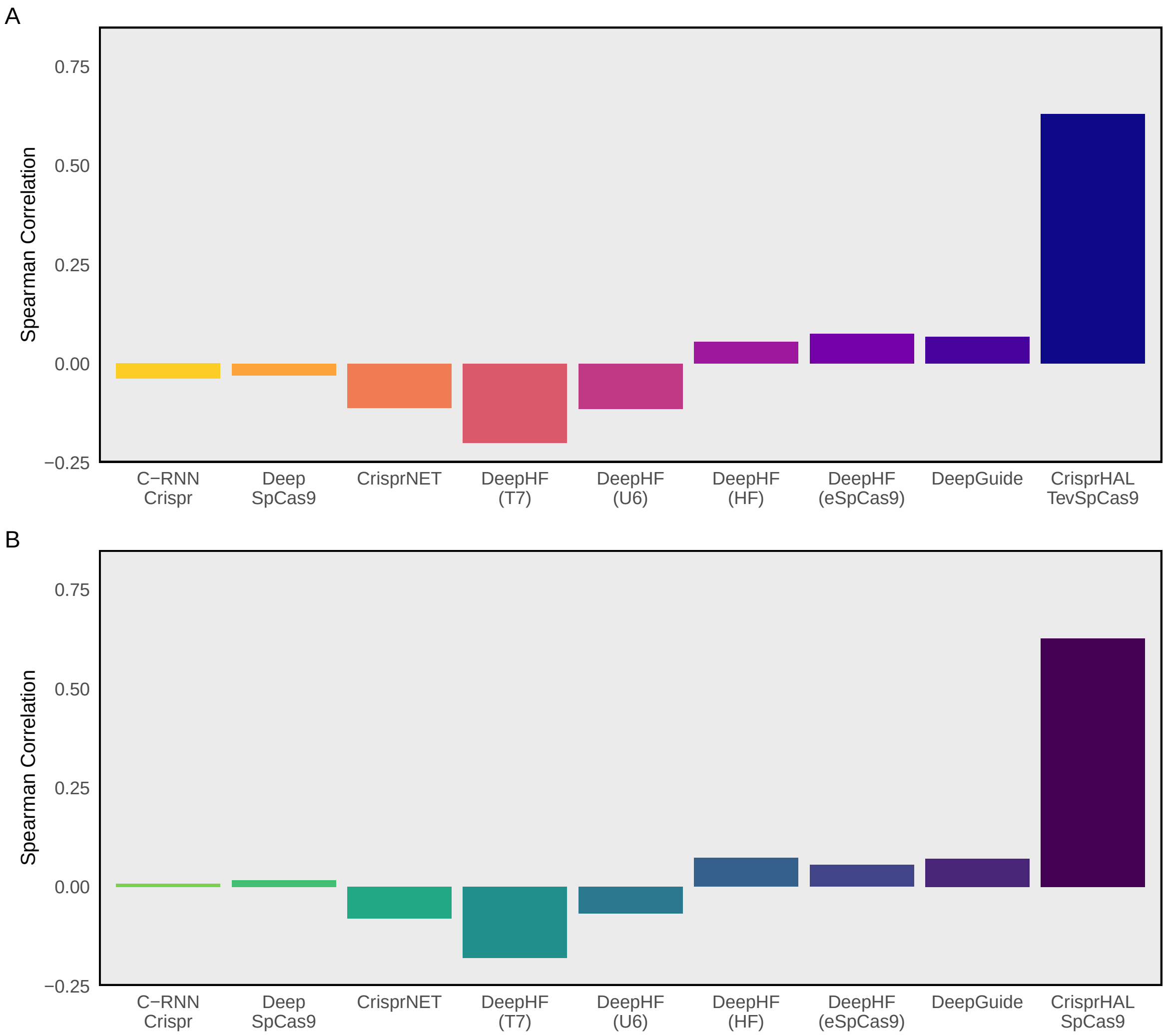

Figure S1: Barcharts are Spearman Rank correlations between the **A)** TevSpCas9 dataset (n=279) and **B)** the SpCas9 dataset (n=303) generated in this study and predictions from eukaryotic sgRNA activity models, compared to the 5-fold cross validation average rank correlation results from the respective version of crisprHAL. The crisprHAL values are reported as the average rank correlation from 5-fold cross validation.

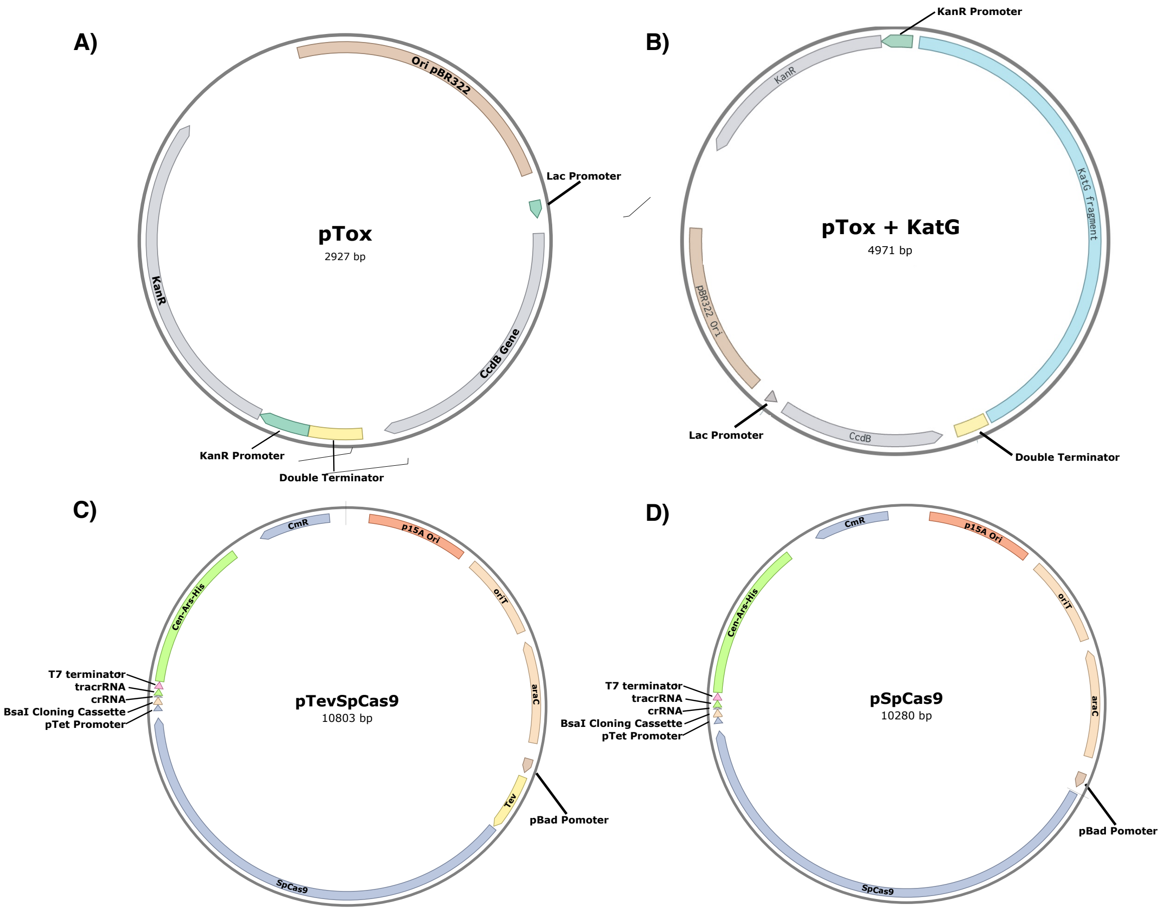

Figure S2: Detailed maps of plasmids used in this study. **A)** The pTox plasmid harbouring the *CcdB* toxic gene under control of Lac promoter; Ori pBR322, medium copy-number origin of replication; KanR, aminoglycoside phosphotransferase gene confers resistance to kanamycin antibiotic. **B)** The same pTox backbone described previously with *Salmonella enterica* LT2 *KatG* gene cloned in. **C)** and **D)** Depiction pTevSpCas9 and pSpCas9 respectively. Each plasmid contains CmR, chloramphenicol acetyl-transferase resistance gene; p15A Ori, medium copy-number origin of replication; OriT, conjugative origin of transfer for incP conjugative systems; araC: L-arabinose regulatory protein responsible for reprssing pBad promoter; pBad promoter, promoter from *E. coli* L-arabinose operon; Tev (pannel **C**), **I**-Tev**I** nuclease domain; SpCas9, Cas9 nuclease from *Streptococcus pyogenes*; sgRNA cassette consisting of: constitutive pTet promoter, Bsa**I** cloning cassette, crRNA and tracrRNA scaffold, and T7 terminator; Cen-Ars-His, centromere and autonomously replicating sequence with histidine biosynthesis gene all of which are required for Yeast Assembly of plasmids. All plasmid maps were created in Benchling (www.benchling.com).

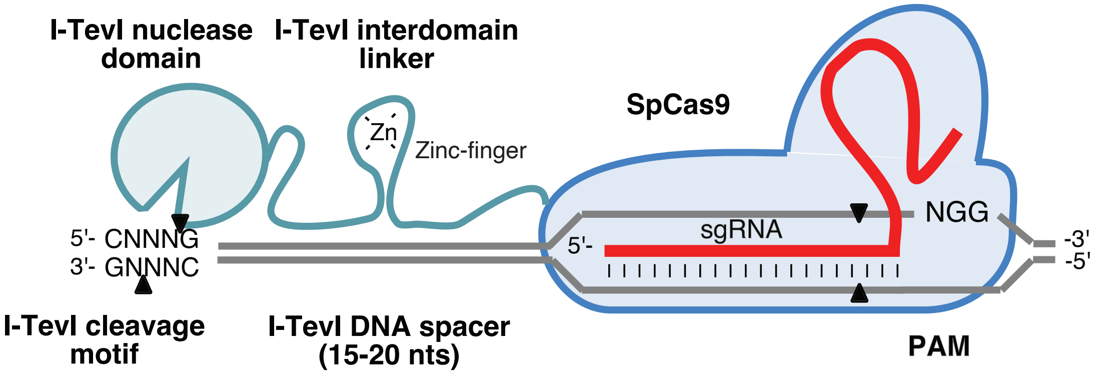

Figure S3: Schematic of TevSpCas9 binding target site. The Cas9 from *Streptococcus pyogenes* binds a target sequence upstream of a 5'-NGG-3' PAM sequence, the flexible linker region allows for recognition and cleavage of I-TevI's cognate target site 5'-CNNNG-3' when it is in 15-20 nucleotides upstream of the end of the sgRNA target.

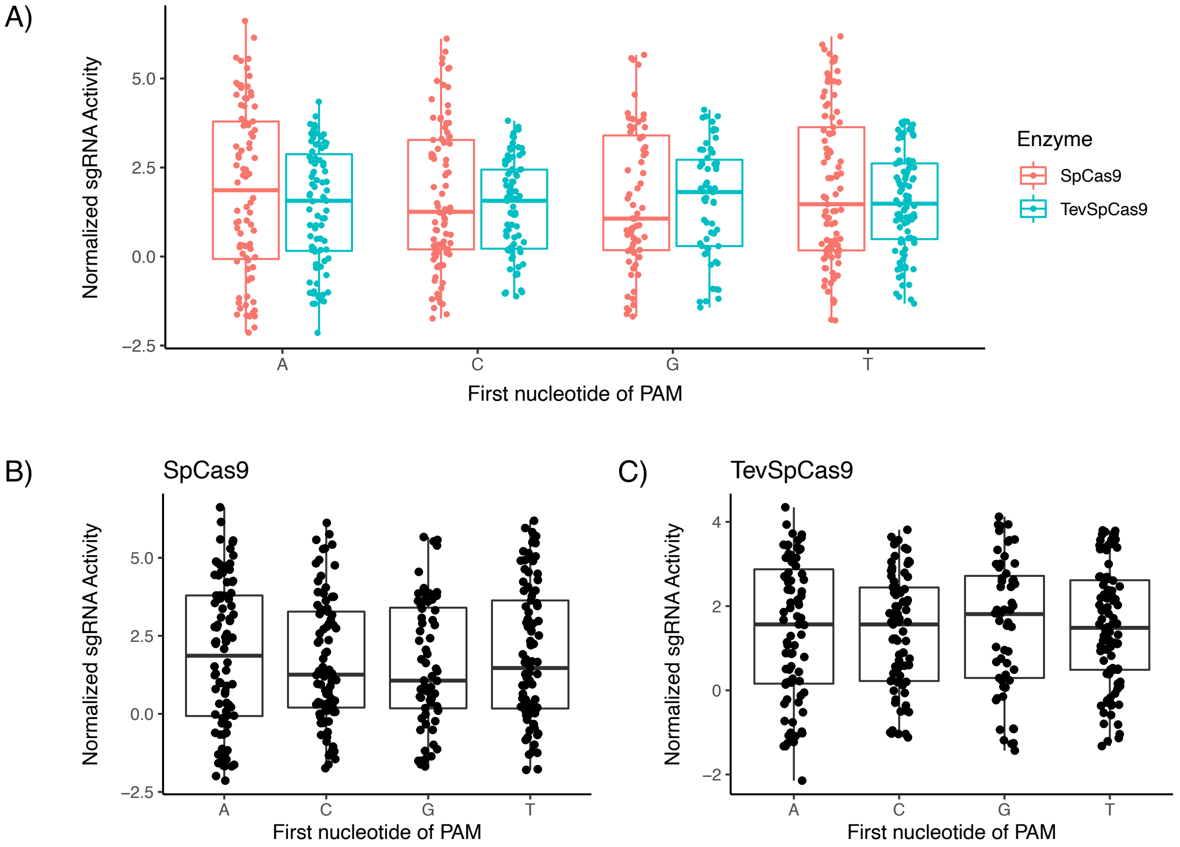

Figure S4: PAM preference plot. **A)** Plot of normalized activity score for sgRNAs in SpCas9 (red) and TevSpCas9 (teal) versus the first nucleotide of the NGG PAM sequence. **B)** and **C)** Similar to previous plot showing SpCas9 (**B**) and TevSpCas9 (**C**) individually.

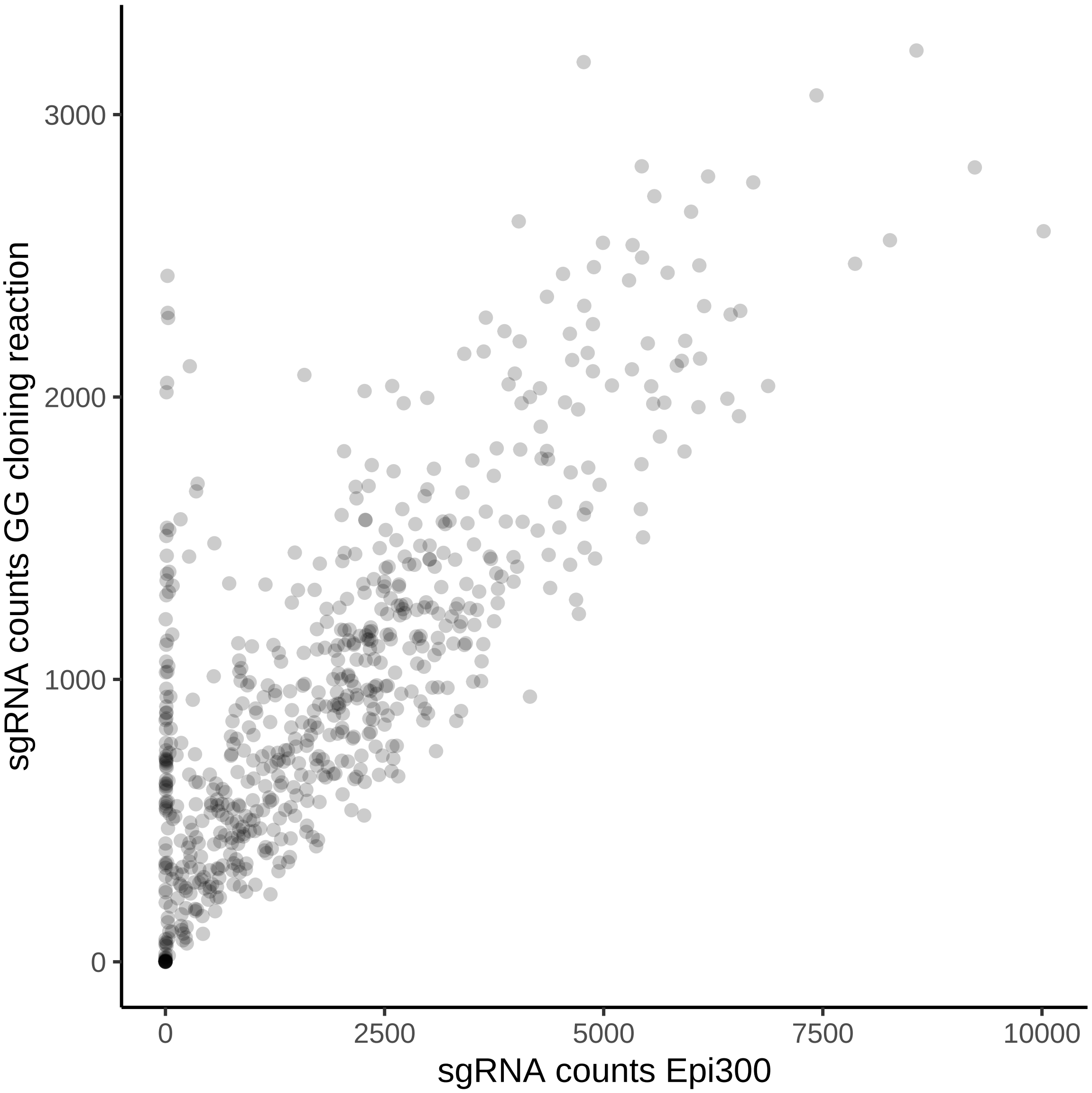

Figure S5: sgRNAs drop out of cloning pool. sgRNAs were cloned into the pSpTevCas9 plasmid and transformed into Epi300 prior to being miniprepped. Illumina primer pairings were used to amplify the pools pre and post-transformation. The y-axis shows the reads counts for sgRNAs in the Golden Gate (GG) cloning reaction prior to being transformed into Epi300. The x-axis shows read counts for the pool isolated from the Epi300 minprep.

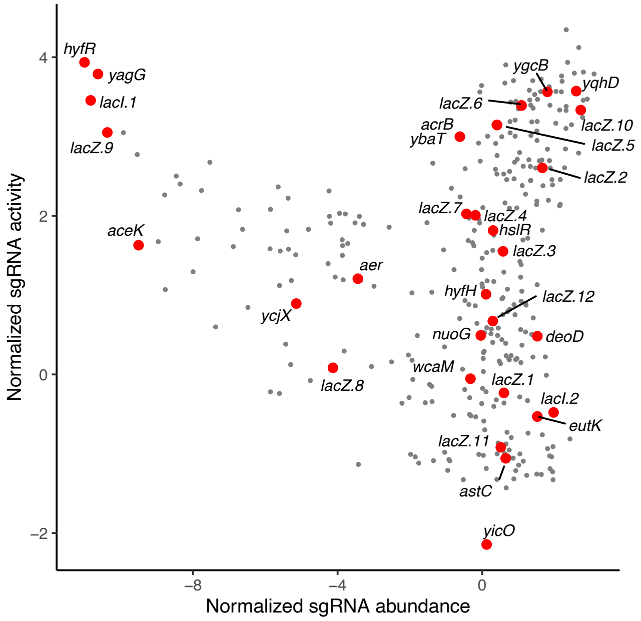

sgRNA ID	sgRNA	Potential off-target	Gene
1602_NC	AAGACACGACTTATCGCCAC	cAGAgcgaACTTATCGCCAC	yqhD
NT30	ACGCGGCTTGGCGAACCGGA	AttCatCgTGGCGAACCGGA	eutK
2059_C	AGCGAATAACAATTTCACAC	AGCGgATAACAATTTCACAC	yicO
2355_C	CACATCCCCCTTTCGCCAGC	CcttcaCCCCTTTCGCCAGC	ybaT
1652_NC	CCCGACCGCTGCGCCTTATC	ggatgCgGCTGCGCCTTATC	ycjX
1640_C	CGCAGCGGTCGGGCTGAACG	gcttattGTCGGGCTGAACG	yagG
2306_C	CGTCGTGACTGGGAAAACCC	gaagaTaACTGGGAAAACCC	hyfH
2385_NC	CTATTACGCCAGCTGGCGAA	gTccgtacCCAGCTGGCGAA	acrB
527_NC	GAATCGAATGCAACCGGCGC	ctggtcgcTGCAACCGGCGC	nuoG
1774_C	TATCCGGTAAGCGGCAGGGT	ggTggGcgAAGCGGCAGGGT	hslR
177_C	ACATGGATGCTGATTTATAT	AacTttcTGCTGATTTATAT	ygcB
1629_C	ACCGGATAAGGCGCAGCGGT	cCaGttTcAGGCGCAGCGGT	aceK
NT13	ATAACGAATGCGCCCGACGC	tTccCcggTGCGCCCGACGC	astC
NT42	ATCGGCCAACGCGCGGGGAG	ATCGGCCAACGCGCGGGGAG	lacI.1
2383_NC	ATTACGCCAGCTGGCGAAAG	gaacaaagAGCTGGCGAAAG	lacZ.1
427_C	CCCCGGGAAAACAGCATTCC	CaCaaaGgAAACAGCATTCC	wcaM
2369_NC	CGAAAGGGGGATGTGCTGCA	tctttaatGGATGTGCTGCA	lacZ.2
2346_NC	CGATTAAGTTGGGTAACGCC	CagcatgaTTGGGTAACGCC	lacZ.3
2435_NC	CGTATAGGCTGCGCAACTGT	CaTtcAGGCTGCGCAACTGT	lacZ.4
1318_NC	CTACGGGGTCTGACGCTCAG	gaAgGtaGTCTGACGCTCAG	deoD
2385_NC	CTATTACGCCAGCTGGCGAA	gTccgtacCCAGCTGGCGAA	lacZ.5
2392_NC	CTCTTCGCTATTACGCCAGC	agggTtatTATTACGCCAGC	lacZ.6
2345_NC	GATTAAGTTGGGTAACGCCA	cATggcGcTGGGTAACGCCA	lacZ.7
1641_C	GCAGCGGTCGGGCTGAACGG	GCAaaaaaCGGGCTGAACGG	hyfR
2541_NC	GGGATCACCATCCGTCGCCC	GatcTCttCATCCGTCGCCC	aer
2434_NC	GTATAGGCTGCGCAACTGTT	aTtcAGGCTGCGCAACTGTT	lacZ.8
NT43	GTCAACCTGCATTAATGAAT	tgCcAgCTGCATTAATGAAT	lacI.2
2417_NC	GTTGGGAAGGGCGATCGGTG	GTTGGGAAGGGCGATCGGTG	lacZ.9
2384_NC	TATTACGCCAGCTGGCGAAA	cAcaggctCAGCTGGCGAAA	lacZ.10
2356_NC	TGCTGCAAGGCGATTAAGTT	aGaTcagtGGCGATTAAGTT	lacZ.11
2382_NC	TTACGCCAGCTGGCGAAAGG	gcgatgatGCTGGCGAAAGG	lacZ.12

Figure S6: Off-target sgRNA predictions and respective activities in pooled assay. Sites in the E. coli genome with complementarity to the seed region of sgRNAs in pooled pTox experiments were identified. These sgRNAs are highlighted in the TevSpCas9 pooled two-plasmid enrichment experiment.

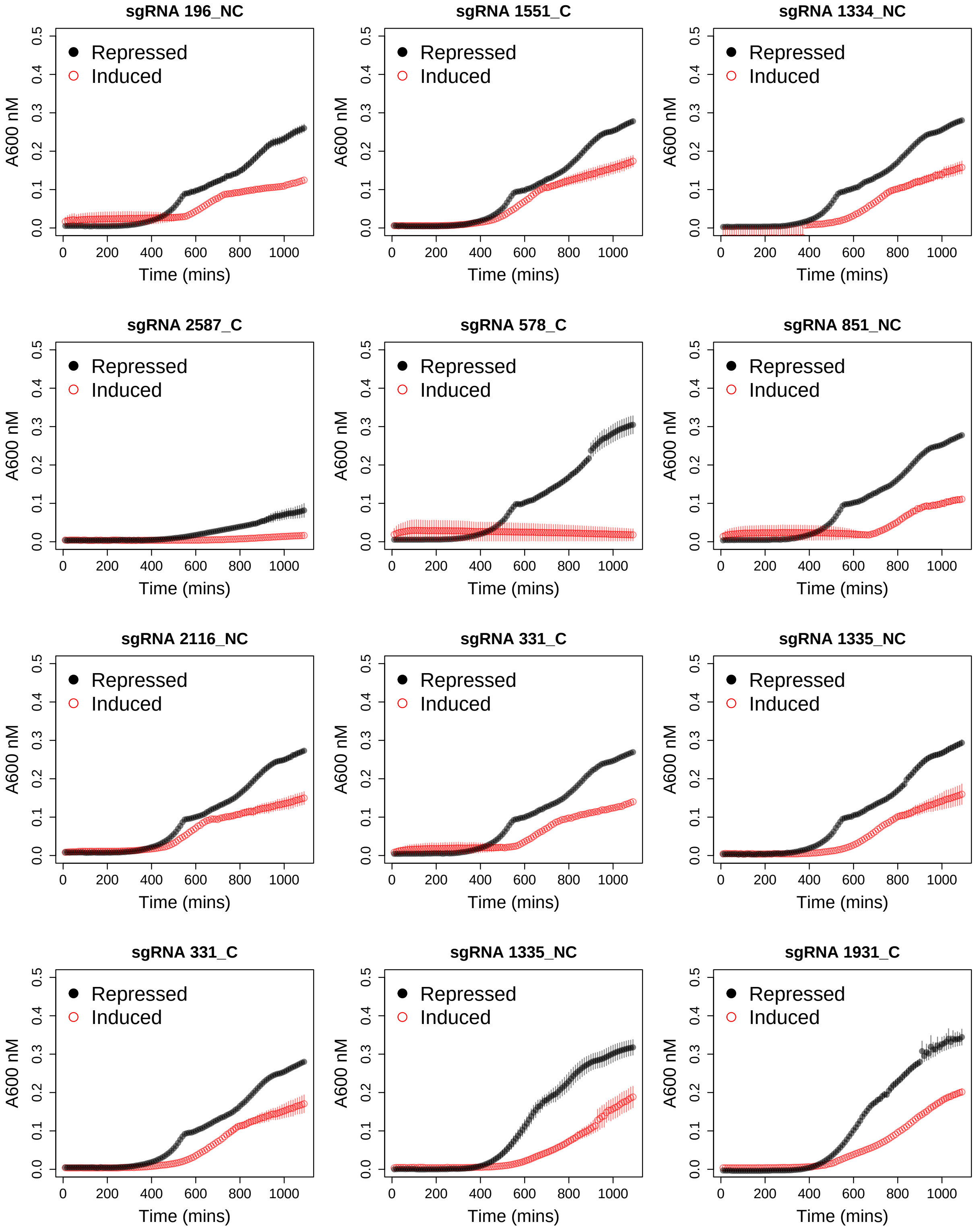
sgRNA 196_NC
Repressed
Induced
A600 nM
Time (mins)
sgRNA 1551_C
Repressed
Induced
A600 nM
Time (mins)
sgRNA 1334_NC
Repressed
Induced
A600 nM
Time (mins)
sgRNA 2587_C
Repressed
Induced
A600 nM
Time (mins)
sgRNA 578_C
Repressed
Induced
A600 nM
Time (mins)
sgRNA 851_NC
Repressed
Induced
A600 nM
Time (mins)
sgRNA 2116_NC
Repressed
Induced
A600 nM
Time (mins)
sgRNA 331_C
Repressed
Induced
A600 nM
Time (mins)
sgRNA 1335_NC
Repressed
Induced
A600 nM
Time (mins)
sgRNA 331_C
Repressed
Induced
A600 nM
Time (mins)
sgRNA 1335_NC
Repressed
Induced
A600 nM
Time (mins)
sgRNA 1931_C
Repressed
Induced
A600 nM
Time (mins)

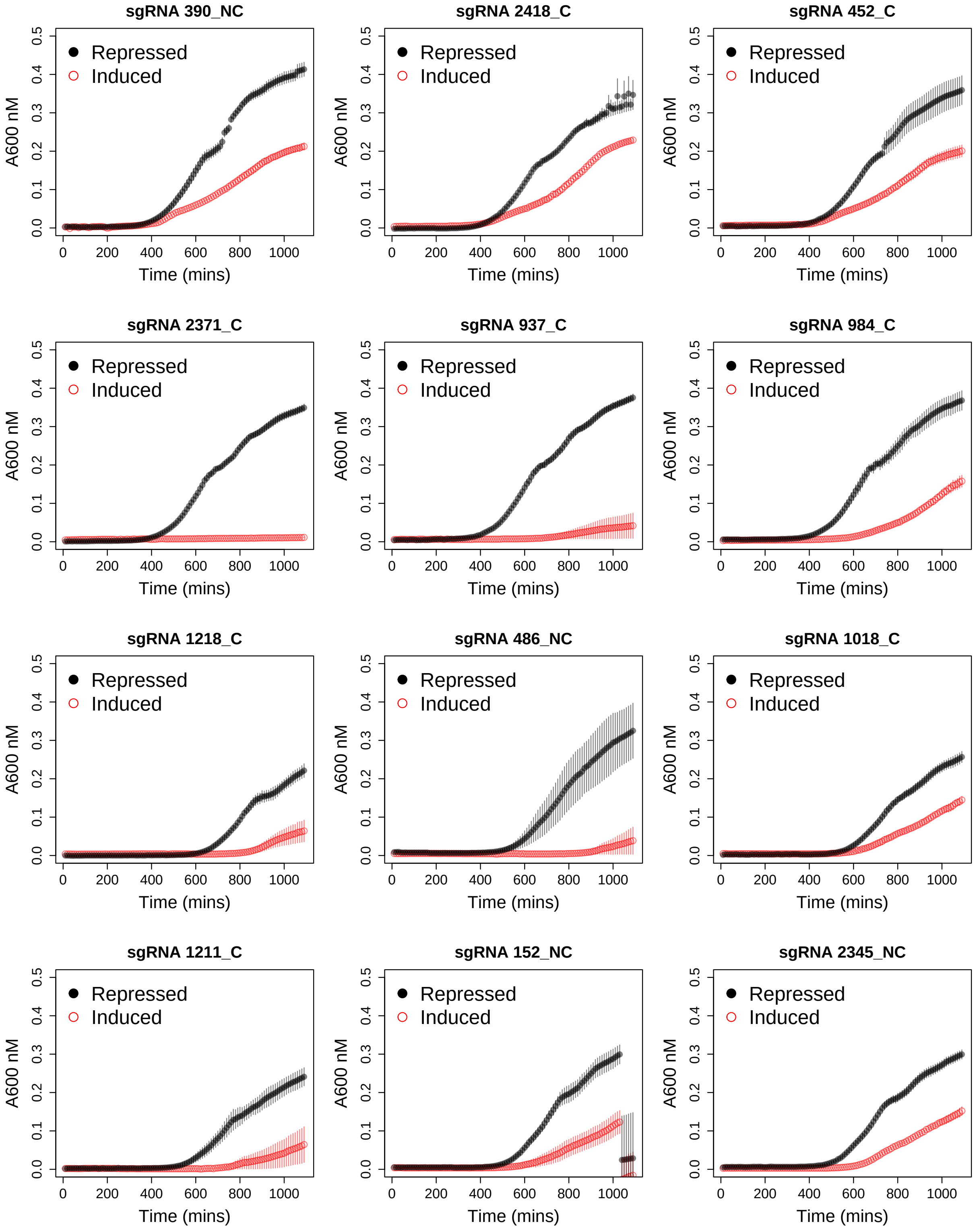
sgRNA 390_NC
sgRNA 2418_C
sgRNA 452_C
sgRNA 2371_C
sgRNA 937_C
sgRNA 984_C
sgRNA 1218_C
sgRNA 486_NC
sgRNA 1018_C
sgRNA 1211_C
sgRNA 152_NC
sgRNA 2345_NC
Repressed
Induced
A600 nM
Time (mins)
0.0
0.1
0.2
0.3
0.4
0.5
0
200
400
600
800
1000

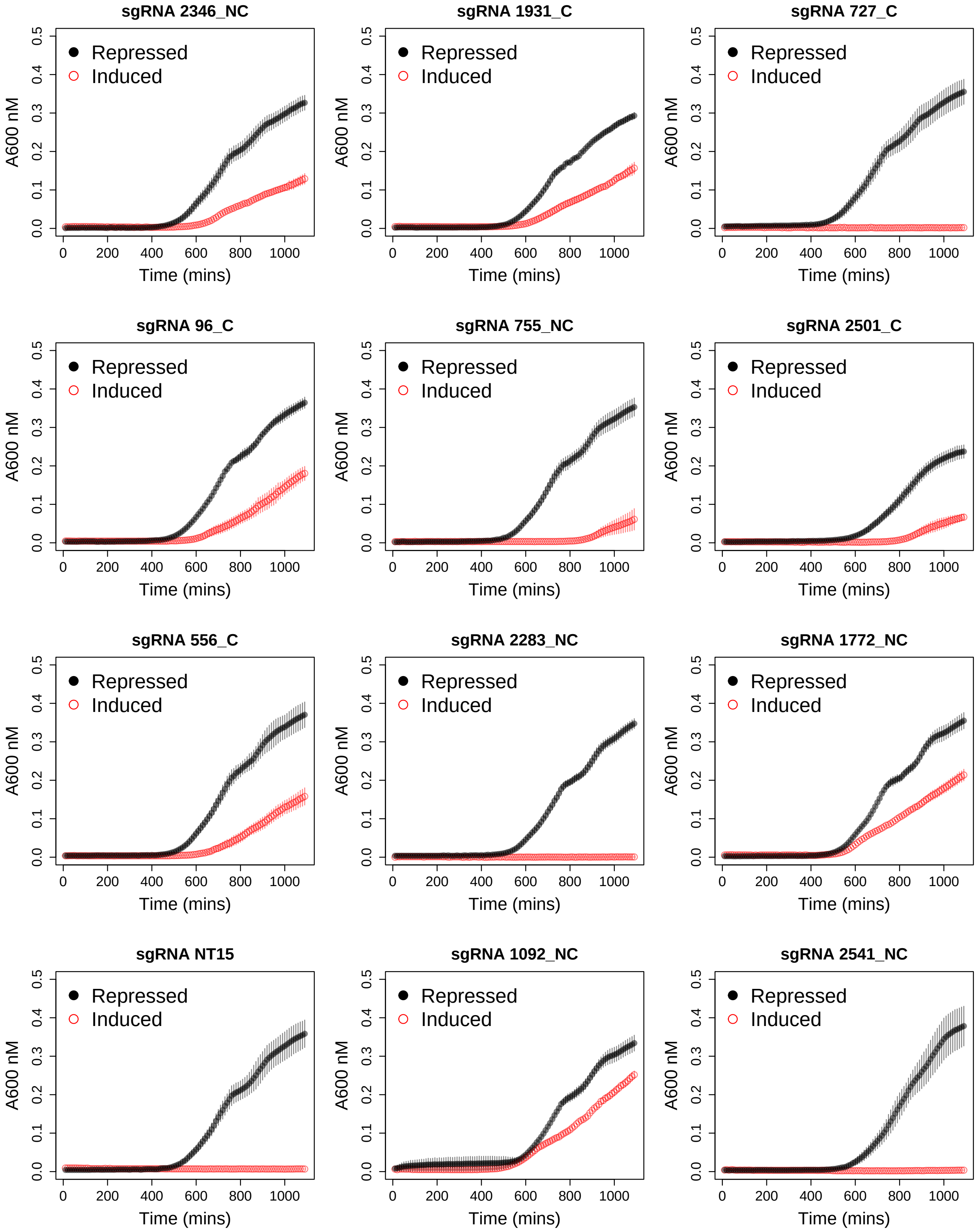
sgRNA 2346_NC
sgRNA 1931_C
sgRNA 727_C
sgRNA 96_C
sgRNA 755_NC
sgRNA 2501_C
sgRNA 556_C
sgRNA 2283_NC
sgRNA 1772_NC
sgRNA NT15
sgRNA 1092_NC
sgRNA 2541_NC
Repressed
Induced
A600 nM
Time (mins)
0.0
0.1
0.2
0.3
0.4
0.5
0
200
400
600
800
1000

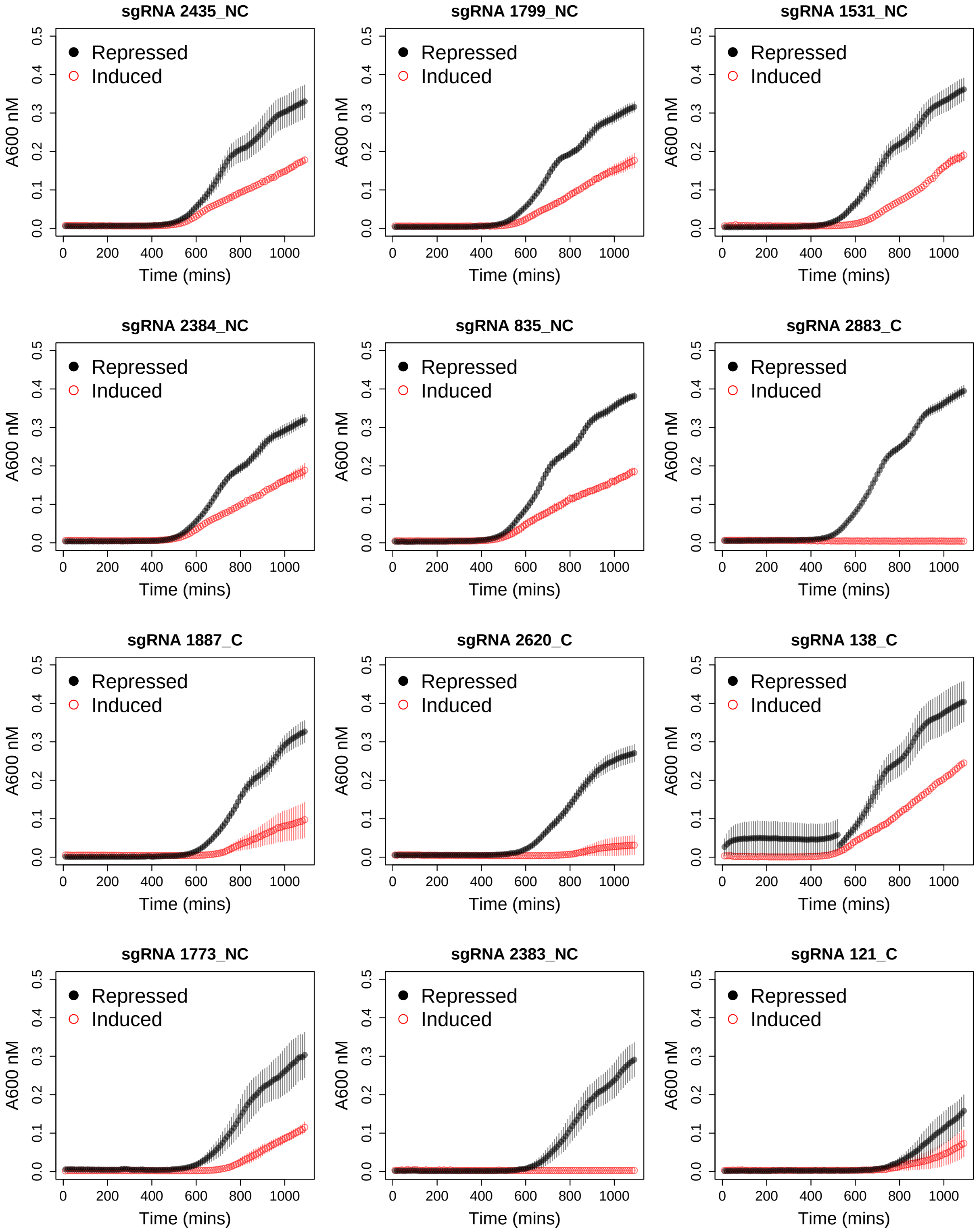
sgRNA 2435_NC
sgRNA 1799_NC
sgRNA 1531_NC
sgRNA 2384_NC
sgRNA 835_NC
sgRNA 2883_C
sgRNA 1887_C
sgRNA 2620_C
sgRNA 138_C
sgRNA 1773_NC
sgRNA 2383_NC
sgRNA 121_C
Repressed
Induced
A600 nM
Time (mins)

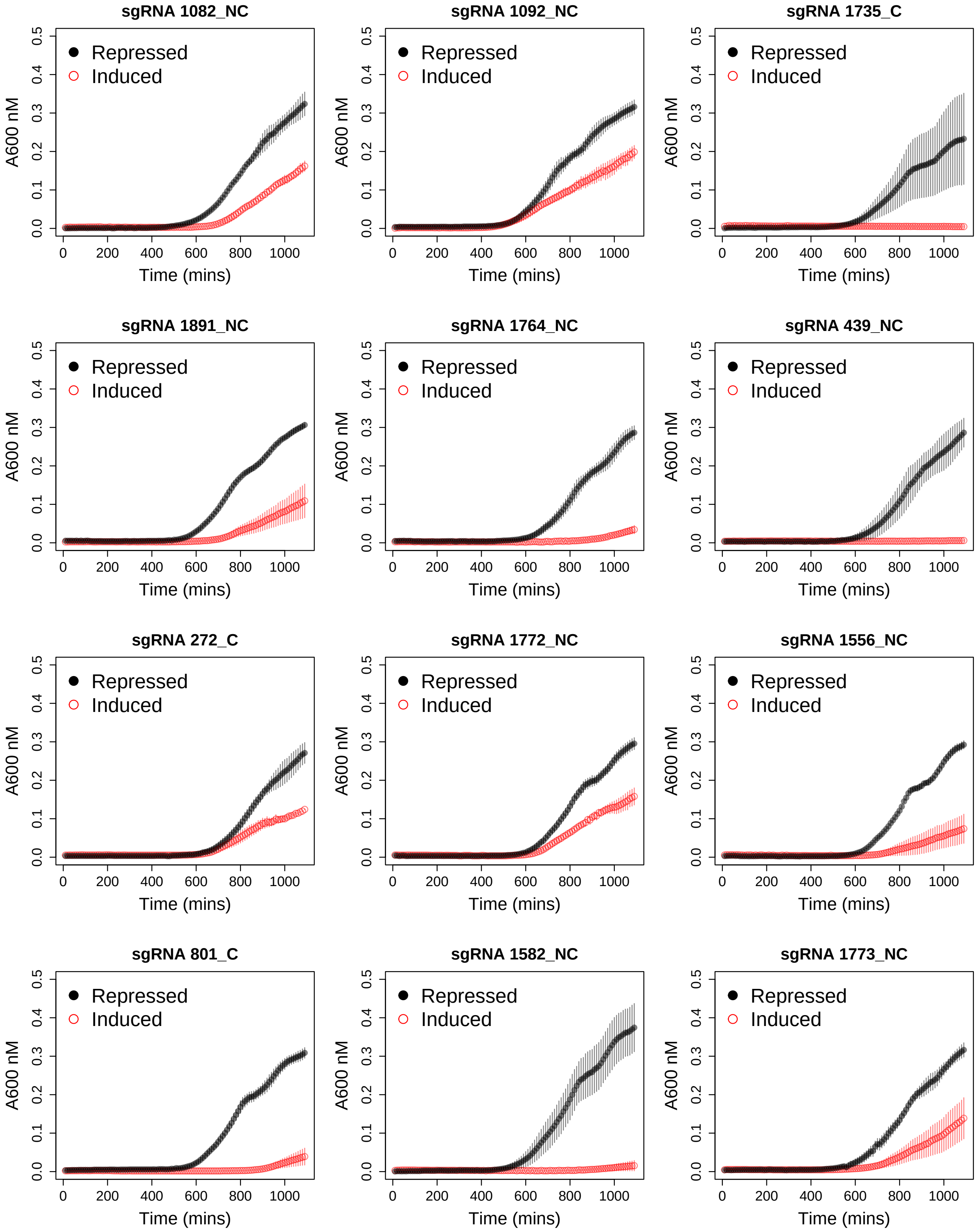
sgRNA 1082_NC
sgRNA 1092_NC
sgRNA 1735_C
sgRNA 1891_NC
sgRNA 1764_NC
sgRNA 439_NC
sgRNA 272_C
sgRNA 1772_NC
sgRNA 1556_NC
sgRNA 801_C
sgRNA 1582_NC
sgRNA 1773_NC
Repressed
Induced
A600 nM
0.0
0.1
0.2
0.3
0.4
0.5
Time (mins)
0
200
400
600
800
1000

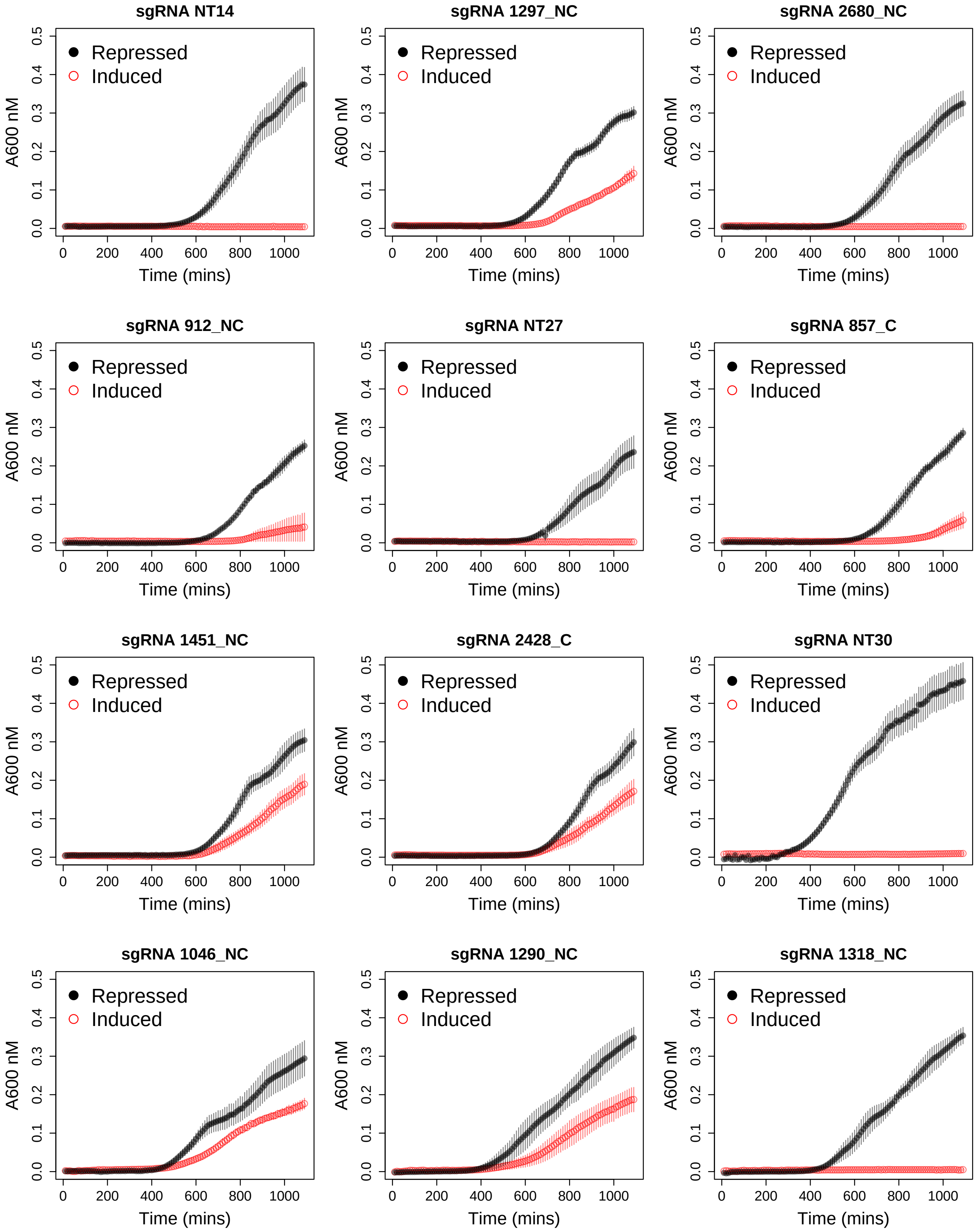
sgRNA NT14
sgRNA 1297_NC
sgRNA 2680_NC
sgRNA 912_NC
sgRNA NT27
sgRNA 857_C
sgRNA 1451_NC
sgRNA 2428_C
sgRNA NT30
sgRNA 1046_NC
sgRNA 1290_NC
sgRNA 1318_NC
Repressed
Induced
A600 nM
Time (mins)
0.0
0.1
0.2
0.3
0.4
0.5
0
200
400
600
800
1000

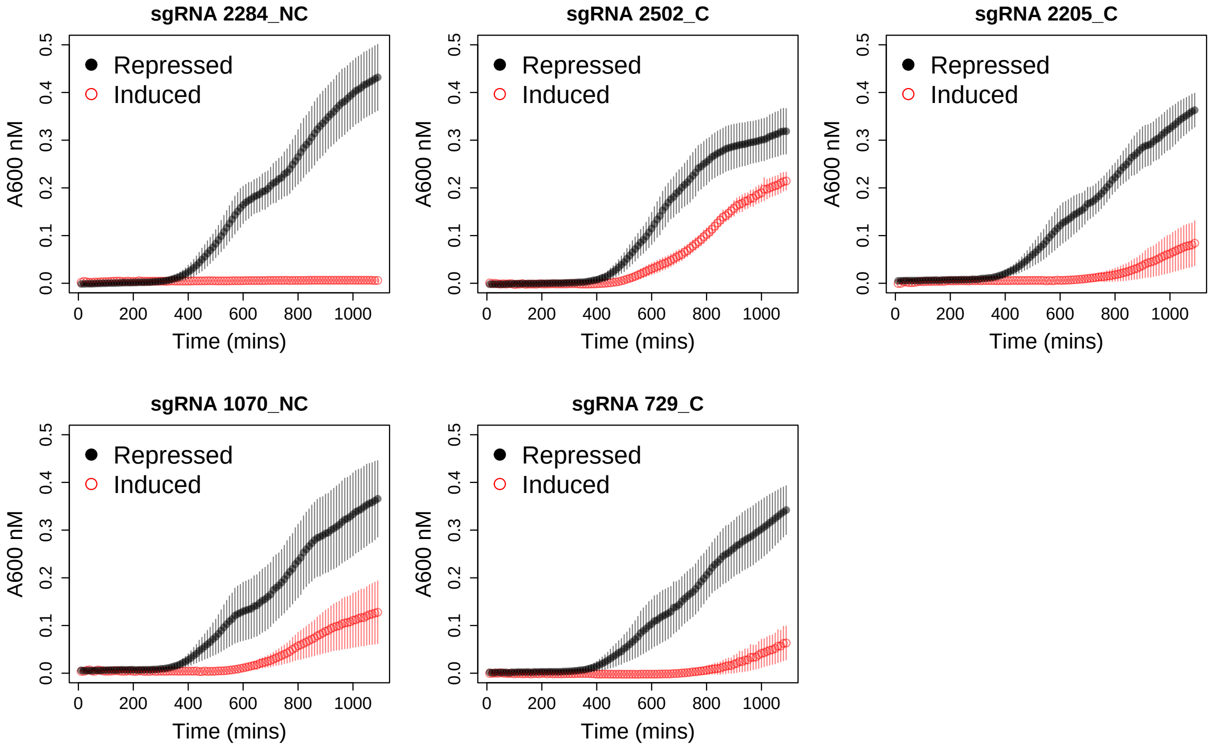

Figure S7: Growth curves for individual sgRNAs targeting pTox. Shown are plots for sgRNAs under induced (red dots and line) and repressed (black dots and line) conditions. Points are the mean of three biological replicates and whiskers represent the mean plus or minus the standard deviation.

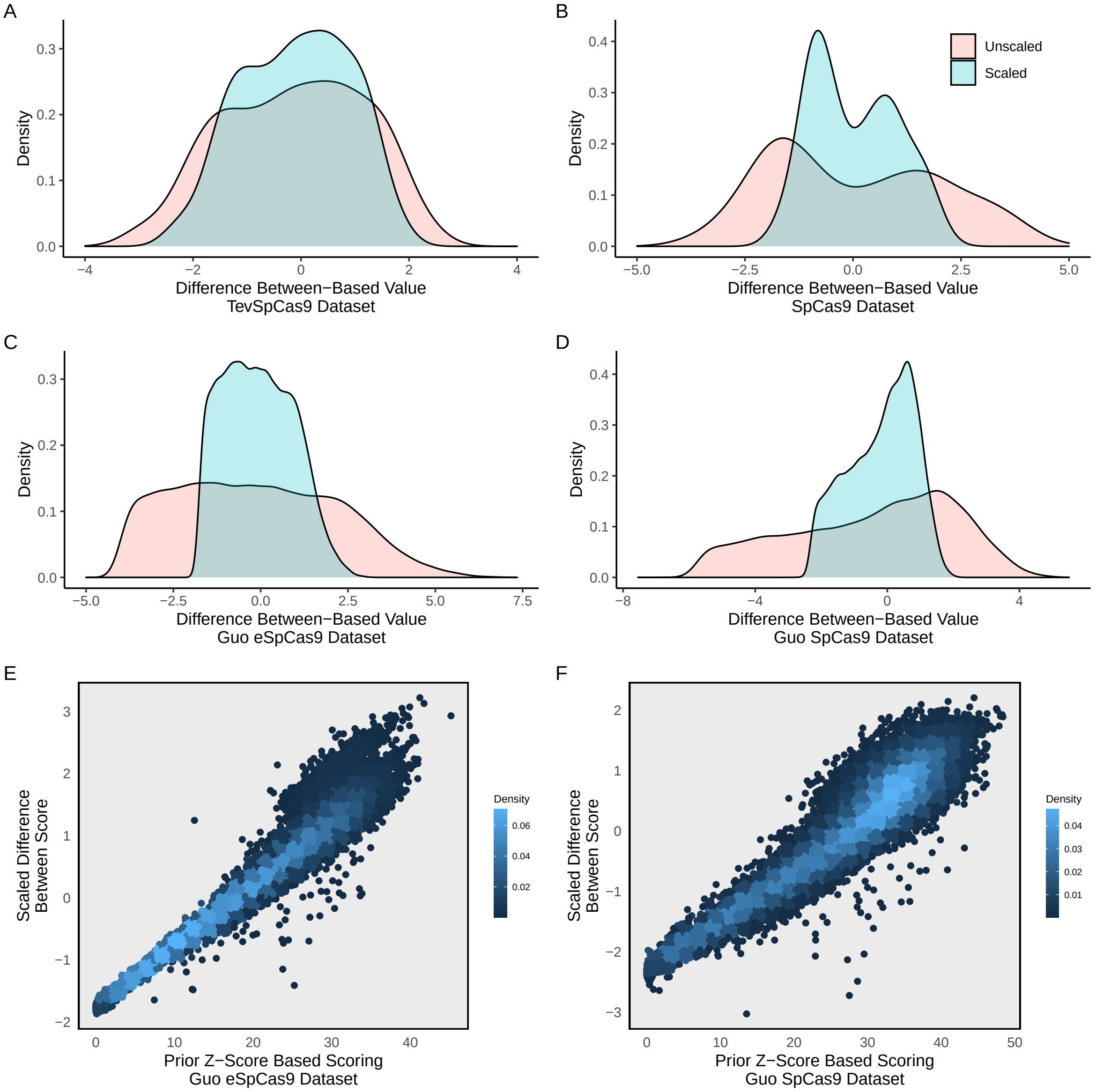

Figure S8: sgRNA associated activity scoring across datasets used in model training. **A-D** Density plots of log-ratio difference between condition activity scores with and without standard deviation scaling for our TevSpCas9 and SpCas9 datasets, and the Guo eSpCas9 and SpCas9 datasets. **E-F** Comparing our scaled activity scores with the original Z-score based activity score values for the Guo eSpCas9 (rank correlation of 0.992) and SpCas9 datasets (rank correlation of 0.938).

sgRNA_ID	sequence	auc_I	auc_R
196_NC	ATATAAATCAGCATCCATGT	1.62	1.00
1551_C	TCTGCTAATCCTGTTACCAG	2.55	1.07
1334_NC	GATCCTTTGATCTTTTCTAC	2.01	1.13
2587_C	TTACCCGGTGGTGCATATCG	0.12	0.24
578_C	GCGCAATCACGAATGAATAA	0.00	1.13
851_NC	ATGAAGGAGAAAACTCACCG	1.14	1.08
2116_NC	TAAATAGCTTGGCGTAATCA	2.24	1.05
331_C	GGTCAGACTAAACTGGCTGA	1.91	1.06
1335_NC	AGATCCTTTGATCTTTTCTA	1.93	1.15
331_C	GGTCAGACTAAACTGGCTGA	2.16	1.09
1335_NC	AGATCCTTTGATCTTTTCTA	1.78	1.35
1931_C	CCAGCAACGCGGCCTTTTTA	2.23	1.39
390_NC	CGGATAAAATGCTTGATGGT	3.03	1.75
2418_C	AGTTGCGCAGCCTATACGTA	2.78	1.40
452_C	TTAGAAGAATATCCTGATTC	2.48	1.43
2371_C	CAGCTGGCGTAATAGCGAAG	0.13	1.44
937_C	CTAAATCAGAATTGGTTAAT	0.36	1.56
984_C	CATTCTGAGAATAGTGTATG	1.34	1.47
1218_C	TTTTTAATTTAAAAGGATCT	0.36	0.59
486_NC	ACAATATTTTCACCTGAATC	0.19	1.02
1018_C	CTCTTGCCCGGCGTCAATAC	1.25	0.81
1211_C	AACTTCATTTTTAATTTAAA	0.43	0.77
152_NC	AAGACGTTTCCCGTTGAATA	1.26	1.09
2345_NC	GATTAAGTTGGGTAACGCCA	1.39	1.05
2346_NC	CGATTAAGTTGGGTAACGCC	1.27	1.17
1931_C	CCAGCAACGCGGCCTTTTTA	1.44	0.99
727_C	TGATAACCTTATTTTTGACG	0.03	1.29
96_C	CTTACATAAACAGTAATACA	1.55	1.27
755_NC	ATTTCCCCTCGTCAAAAATA	0.29	1.21
2501_C	AGTGATATTATTGACACGCC	0.44	0.71
556_C	TCGCGTATTTCGTCTCGCTC	1.32	1.26
2283_NC	TTGTAATACGACTTCGAATA	0.00	1.10
1772_NC	CTGTCCGCCTTTCTCCCTTC	2.25	1.20
NT15	ATACGGTTATCCACAGAATC	0.00	1.20
1092_NC	AATGCTCCTATACGACGTTT	2.54	1.12
2541_NC	GGGATCACCATCCGTCGCCC	0.00	1.07
2435_NC	CGTATAGGCTGCGCAACTGT	1.90	1.12
1799_NC	GTTCCGACCCTGCCGCTTAC	1.84	1.10
1531_NC	CTACAGAGTTCTTGAAGTGG	1.64	1.25
2384_NC	TATTACGCCAGCTGGCGAAA	2.09	1.10
835_NC	ACCGAGGCAGTTCCATAGGA	2.26	1.43
2883_C	GAGTCACACTGGCTCACCTT	0.04	1.41
1887_C	ATTTTTGTGATGCTCGTCAG	0.77	0.93
2620_C	GCGCATGATGACCACCGATA	0.22	0.81
138_C	AACGGGAAACGTCTTGCTCG	2.64	1.42
1773_NC	CCTGTCCGCCTTTCTCCCTT	0.80	0.85
2383_NC	ATTACGCCAGCTGGCGAAAG	0.02	0.73
121_C	TGTTATGAGCCATATTCAAC	0.39	0.29
1082_NC	TACGACGTTTAGGCTATAAA	1.22	0.91
1092_NC	AATGCTCCTATACGACGTTT	2.24	1.03
1735_C	GAAAGCGCCACGCTTCCCGA	0.04	0.69
1891_NC	AAAATCGACGCTCAAGTCAG	0.82	0.93
1764_NC	CTTTCTCCCTTCGGGAAGCG	0.19	0.72
439_NC	TGTTTTCCCGGGGATCGCAG	0.02	0.73
272_C	CCAGAGTTGTTTCTGAAACA	1.09	0.64

sgRNA_ID	sequence	auc_I	auc_R
1772_NC	CTGTCCGCCTTTCTCCCTTC	1.44	0.79
1556_NC	AGCAGAGCGAGGTATGTAGG	0.51	0.76
801_C	ACCAGGATCTTGCCATCCTA	0.17	0.92
1582_NC	TGGCAGCAGCCACTGGTAAC	0.10	1.13
1773_NC	CCTGTCCGCCTTTCTCCCTT	0.97	0.86
NT14	ACGGTTATCCACAGAATCAG	0.04	1.08
1297_NC	GGAACGAAAACTCACGTTAA	1.06	0.93
2680_NC	AGCCACTTCTTCCCCGATAA	0.02	0.96
912_NC	AACTGCAATTTATTCATATC	0.27	0.62
NT27	GTTGGCCGATTCATTAATGC	0.04	0.58
857_C	AAACGGCTTTTTCAAAAATA	0.27	0.70
1451_NC	GTTACCTTCGGAAAAAGAGT	1.58	0.83
2428_C	CCTATACGTACGGCAGTTTA	1.30	0.69
NT30	ACGCGGCTTGGCGAACCGGA	0.00	0.88
1046_NC	AAGCATCCCGTATTGACGCC	0.85	0.68
1290_NC	AAACTCACGTTAAGGGATTT	0.65	0.78
1318_NC	CTACGGGGTCTGACGCTCAG	0.00	0.62
2284_NC	ATTGTAATACGACTTCGAAT	0.04	0.97
2502_C	GTGATATTATTGACACGCCG	1.52	0.98
2205_C	GCAGATATCCATCACACTGG	0.04	0.75
1070_NC	GCTATAAAAGGTTGAATAAG	0.04	0.97
729_C	ATAACCTTATTTTTGACGAG	0.00	0.73

Table S1: Area under the curve (AUC) calcuations for induced (auc_I) and repressed (auc_R) conditions for each sgRNA tested.

crRNA	DNA	Position	Direction	Mismatches
ATTACGCCAGCTGGCGAAAGNGG	cTTtttCCAGCTGGCGAAtGTGG	459570	+	5
ATTACGCCAGCTGGCGAAAGNGG	ATgACGCCtGCTGcCGcgAGTGG	1442414	+	5
ATTACGCCAGCTGGCGAAAGNGG	ATTAgGCgAtCTGGtGAAAGAGG	1628762	+	4
ATTACGCCAGCTGGCGAAAGNGG	gaTACGCCAGCcaGCGAAAaTGG	2609869	-	5
ATTACGCCAGCTGGCGAAAGNGG	ATTACGCtgGCTGGaGAtAtAGG	2613786	-	5
**ATTACGCCAGCTGGCGAAAGNGG	ATTACGCCAGCTGGCGAAAGGGG	4275441	-	0
ATTACGCCAGCTGGCGAAAGNGG	ATTACGCCAGCaGGaaAAgtGGG	4136342	-	5
GAAAGCGCCACGCTTCCCGANGG	GAAAGaGCCACGCggCgtGATGG	784312	+	5
GAAAGCGCCACGCTTCCCGANGG	GAtAGCGaCgCcaTTCCCGACGG	4172727	+	5
GAAAGCGCCACGCTTCCCGANGG	GAAAGCGCCgCGCgcCaCGcCGG	4421779	+	5
TGTTTTCCCGGGGATCGCAGNGG	TcTcTTCCtGGGGtaCGCAGTGG	1142981	+	5
TGTTTTCCCGGGGATCGCAGNGG	cGTTTTCCCaGcttTCGCAGCGG	1968723	+	5
TGTTTTCCCGGGGATCGCAGNGG	gGaTTTCCCGGGGAgaGCtGAGG	2566136	-	5
TGTTTTCCCGGGGATCGCAGNGG	TaTTTTCCCGGGcATCGgccTGG	3087377	+	5
AGCCACTTCTTCCCCGATAANGG	AaaCACaTcGTcGcCCGATAATGG	727635	-	5
AGCCACTTCTTCCCCGATAANGG	AtCgAaTcCTTCCtCGATAAAGG	1327345	+	5
AGCCACTTCTTCCCCGATAANGG	AaCCACTTtTTCCgCaATAgCGG	1493689	+	5
AGCCACTTCTTCCCCGATAANGG	AGCCgCgTCTTtgCCGATAcCGG	4053092	+	5
AGCCACTTCTTCCCCGATAANGG	ccCCAgTTCTTCgCCGATAATGG	4542326	+	4
GTTGGCCGATTCATTAATGCNGG	aTTGGCtGAaTCATTAAcGgTGG	739500	+	5
GTTGGCCGATTCATTAATGCNGG	GgTGGCtGAaTCATTAATGgCGG	3333486	+	4
GTTGGCCGATTCATTAATGCNGG	aaTGGCgctTTCATTAATGCTGG	3084823	-	5
GTTGGCCGATTCATTAATGCNGG	GcTGGCaGtTTCgcTAATGCTGG	3996016	+	5
GTTGGCCGATTCATTAATGCNGG	tTTGGgCaAaTCATTAAcGCCGG	4570844	-	5
ACGCGGCTTGGCGAACCGGANGG	gCGCtGCgTGaCGAACtGGACGG	363831	-	5
ACGCGGCTTGGCGAACCGGANGG	gCGCaGtTTGGCGtACCGGcGGG	2362003	+	5
ACGCGGCTTGGCGAACCGGANGG	ACGCaGCgTGaaGgACCGGAAGG	2615366	-	5
ACGCGGCTTGGCGAACCGGANGG	ACGCGGCTTcGCGAAtttGcGGG	3619565	-	5
ACGCGGCTTGGCGAACCGGANGG	cCGCaGCTTcGCGcAgCGGATGG	4015712	+	5
CTACGGGGTCTGACGCTCAGNGG	gaAgGtaGTCTGACGCTCAGCGG	20104	+	5
ATTGTAATACGACTTCGAATNGG	ATTGTAtaACGcCaTtGAATGGG	4269141	-	5
GCAGATATCCATCACACTGGNGG	GgAGAgATCaATCcCACcGGTGG	188827	+	5
GCAGATATCCATCACACTGGNGG	GCcGATATCCtgCggACTGGCGG	649252	+	5
GCAGATATCCATCACACTGGNGG	GggaATATCCATCAtACTGGTGG	781001	+	4
GCAGATATCCATCACACTGGNGG	GCgGATAaCCATCACgCaGcAGG	3434873	+	5
GCAGATATCCATCACACTGGNGG	GCAGATcgCCgcCgCACTGGCGG	3565130	+	5
GCAGATATCCATCACACTGGNGG	GCAGtcATCCggCACgCTGGTGG	3729093	-	5
GCAGATATCCATCACACTGGNGG	GCAGtaATCCAgCAaACTGaCGG	3895243	+	5
GCAGATATCCATCACACTGGNGG	GCgGATATCCggCgCACcGGGGG	4047183	-	5
GCAGATATCCATCACACTGGNGG	GCAaAaATCCATttCgCTGGTGG	4277571	-	5
ATAACCTTATTTTTGACGAGNGG	ATAcCtTTATTgTTGgaGAGGGG	2703519	+	5
ATAACCTTATTTTTGACGAGNGG	ATAAtCTgATTggTGACGtGCGG	3167229	+	5
ATAACCTTATTTTTGACGAGNGG	AaAAttTTgTTTTTGACGAGCGG	3524084	-	4

Table S2: Off-targets in the *E. coli* K-12 genome for sgRNAs classified as toxic in growth curves. Please note that crRNA marked with two asterisks (**) is an exact match to the LacZ alpha fragment coding sequence that is deleted in our *E. coli* strain and therefore is not an off-target.

Name	Sequence (5’ to 3’)	Notes
Illumina_fwd_1	ACACTCTTTCCCTACACGACGCTC<br>TTCCGATCTnnnnTGCATACACTGG<br>AGTGATAGAGATACTGAGCACG	Forward<br>primer 12 mer bar-<br>code(TGCATACACTGG)
Illumina_fwd_2	ACACTCTTTCCCTACACGACGCTC<br>TTCCGATCTnnnnACTCACAGGAAT<br>AGTGATAGAGATACTGAGCACG	Forward<br>primer 12 mer bar-<br>code(ACTCACAGGAAT)
Illumina_fwd_3	ACACTCTTTCCCTACACGACGCTC<br>TTCCGATCTnnnnGTAGGTGCTTAC<br>AGTGATAGAGATACTGAGCACG	Forward<br>primer 12 mer bar-<br>code(GTAGGTGCTTAC)
Illumina_fwd_4	ACACTCTTTCCCTACACGACGCTC<br>TTCCGATCTnnnnCAGTCGTTAAGA<br>AGTGATAGAGATACTGAGCACG	Forward<br>primer 12 mer bar-<br>code(CAGTCGTTAAGA)
Illumina_fwd_5	ACACTCTTTCCCTACACGACGCTC<br>TTCCGATCTnnnnCACTACGCTAGA<br>AGTGATAGAGATACTGAGCACG	Forward<br>primer 12 mer bar-<br>code(CACTACGCTAGA)
Illumina_fwd_6	ACACTCTTTCCCTACACGACGCTC<br>TTCCGATCTnnnnGCTCGAAGATTC<br>AGTGATAGAGATACTGAGCACG	Forward<br>primer 12 mer bar-<br>code(GCTCGAAGATTC)
Illumina_fwd_7	ACACTCTTTCCCTACACGACGCTC<br>TTCCGATCTnnnnTGAACGTTGGAT<br>AGTGATAGAGATACTGAGCACG	Forward<br>primer 12 mer bar-<br>code(TGAACGTTGGAT)
Illumina_fwd_8	ACACTCTTTCCCTACACGACGCTC<br>TTCCGATCTnnnnATGGTTCACCCG<br>AGTGATAGAGATACTGAGCACG	Forward<br>primer 12 mer bar-<br>code(ATGGTTCACCCG)
Illumina_fwd_9	ACACTCTTTCCCTACACGACGCTC<br>TTCCGATCTnnnnCGAGGGAAAGTC<br>AGTGATAGAGATACTGAGCACG	Forward<br>primer 12 mer bar-<br>code(CGAGGGAAAGTC)
Illumina_fwd_10	ACACTCTTTCCCTACACGACGCTC<br>TTCCGATCTnnnnTACTACGTGGCC<br>AGTGATAGAGATACTGAGCACG	Forward<br>primer 12 mer bar-<br>code(TACTACGTGGCC)
Illumina_fwd_11	ACACTCTTTCCCTACACGACGCTC<br>TTCCGATCTnnnnGTTCCTCCATTAA<br>GTGATAGAGATACTGAGCACG	Forward<br>primer 12 mer bar-<br>code(GTTCCTCCATTA)
Illumina_fwd_12	ACACTCTTTCCCTACACGACGCTC<br>TTCCGATCTnnnnACGATATGGTCA<br>AGTGATAGAGATACTGAGCACG	Forward<br>primer 12 mer bar-<br>code(ACGATATGGTCA)
Illumina_fwd_13	ACACTCTTTCCCTACACGACGCTC<br>TTCCGATCTnnnnTATCGACACAAG<br>AGTGATAGAGATACTGAGCACG	Forward<br>primer 12 mer bar-<br>code(TATCGACACAAG)
Illumina_fwd_14	ACACTCTTTCCCTACACGACGCTC<br>TTCCGATCTnnnnAGCATGTCCCGT<br>AGTGATAGAGATACTGAGCACG	Forward<br>primer 12 mer bar-<br>code(AGCATGTCCCGT)
Illumina_fwd_15	ACACTCTTTCCCTACACGACGCTC<br>TTCCGATCTnnnnCCAGATATAGCA<br>AGTGATAGAGATACTGAGCACG	Forward<br>primer 12 mer bar-<br>code(CCAGATATAGCA)
Illumina_fwd_16	ACACTCTTTCCCTACACGACGCTC<br>TTCCGATCTnnnnGTGTCCGGATTC<br>AGTGATAGAGATACTGAGCACG	Forward<br>primer 12 mer bar-<br>code(GTGTCCGGATTC)
Illumina_fwd_17	ACACTCTTTCCCTACACGACGCTC<br>TTCCGATCTnnnnATCGCACAGTAA<br>AGTGATAGAGATACTGAGCACG	Forward<br>primer 12 mer bar-<br>code(ATCGCACAGTAA)
Illumina_fwd_18	ACACTCTTTCCCTACACGACGCTC<br>TTCCGATCTnnnnCAGCTCATCAGC<br>AGTGATAGAGATACTGAGCACG	Forward<br>primer 12 mer bar-<br>code(CAGCTCATCAGC)

Name	Sequence (5' to 3')	Notes
Illumina_fwd_19	ACACTCTTTCCCTACACGACGCTCTTCCGATCTnnnnGCATATGCACTGAGTGATAGAGATACTGAGCACG	Forward primer 12 mer bar-code(GCATATGCACTG)
Illumina_fwd_20	ACACTCTTTCCCTACACGACGCTCTTCCGATCTnnnnTGTAGGTGTGCTAGTGATAGAGATACTGAGCACG	Forward primer 12 mer bar-code(TGTAGGTGTGCT)
Illumina_fwd_21	ACACTCTTTCCCTACACGACGCTCTTCCGATCTnnnnACGAGACTGATTAGTGATAGAGATACTGAGCACG	Forward primer 12 mer bar-code(ACGAGACTGATT)
Illumina_rev_1	CGGTCTCGGCATTCCTGCTGAACCGCTCTTCCGATCTnnnnTGCATACACTGGGCCTTATTTTAACTTGCTATTTCTAGCTC	Reverse primer 12 mer bar-code(TGCATACACTGG)
Illumina_rev_2	CGGTCTCGGCATTCCTGCTGAACCGCTCTTCCGATCTnnnnACTCACAGGAATGCCTTATTTTAACTTGCTATTTCTAGCTC	Reverse primer 12 mer bar-code(ACTCACAGGAAT)
Illumina_rev_3	CGGTCTCGGCATTCCTGCTGAACCGCTCTTCCGATCTnnnnGTAGGTGCTTACGCCTTATTTTAACTTGCTATTTCTAGCTC	Reverse primer 12 mer bar-code(GTAGGTGCTTAC)
Illumina_rev_4	CGGTCTCGGCATTCCTGCTGAACCGCTCTTCCGATCTnnnnCAGTCGTTAAGAGCCTTATTTTAACTTGCTATTTCTAGCTC	Reverse primer 12 mer bar-code(CAGTCGTTAAGA)
Illumina_rev_5	CGGTCTCGGCATTCCTGCTGAACCGCTCTTCCGATCTnnnnCACTACGCTAGAGCCTTATTTTAACTTGCTATTTCTAGCTC	Reverse primer 12 mer bar-code(CACTACGCTAGA)
Illumina_rev_6	CGGTCTCGGCATTCCTGCTGAACCGCTCTTCCGATCTnnnnGCTCGAAGATTCGCCTTATTTTAACTTGCTATTTCTAGCTC	Reverse primer 12 mer bar-code(GCTCGAAGATTC)
Illumina_rev_7	CGGTCTCGGCATTCCTGCTGAACCGCTCTTCCGATCTnnnnTGAACGTTGGATGCCTTATTTTAACTTGCTATTTCTAGCTC	Reverse primer 12 mer bar-code(TGAACGTTGGAT)
Illumina_rev_8	CGGTCTCGGCATTCCTGCTGAACCGCTCTTCCGATCTnnnnATGGTTCACCCGGCCTTATTTTAACTTGCTATTTCTAGCTC	Reverse primer 12 mer bar-code(ATGGTTCACCCG)
Illumina_rev_9	CGGTCTCGGCATTCCTGCTGAACCGCTCTTCCGATCTnnnnCGAGGGAAAGTCGCCTTATTTTAACTTGCTATTTCTAGCTC	Reverse primer 12 mer bar-code(CGAGGGAAAGTC)
Illumina_rev_10	CGGTCTCGGCATTCCTGCTGAACCGCTCTTCCGATCTnnnnTACTACGTGGCCGCCTTATTTTAACTTGCTATTTCTAGCTC	Reverse primer 12 mer bar-code(TACTACGTGGCC)
Illumina_rev_11	CGGTCTCGGCATTCCTGCTGAACCGCTCTTCCGATCTnnnnGTTCCTCCATTAGCCTTATTTTAACTTGCTATTTCTAGCTC	Reverse primer 12 mer bar-code(GTTCCTCCATTA)

Name	Sequence (5' to 3')	Notes
Illumina_rev_12	CGGTCTCGGCATTCCTGCTGAACCGCTCTTCCGATCTnnnnACGATATGGTCAGCCTTATTTTAACTTGCTATTTCTAGCTC	Reverse primer 12 mer bar-code(ACGATATGGTCA)
Illumina_rev_13	CGGTCTCGGCATTCCTGCTGAACCGCTCTTCCGATCTnnnnTATCGACACAAGGCCTTATTTTAACTTGCTATTTCTAGCTC	Reverse primer 12 mer bar-code(TATCGACACAAG)
Illumina_rev_14	CGGTCTCGGCATTCCTGCTGAACCGCTCTTCCGATCTnnnnAGCATGTCCCGTGCCTTATTTTAACTTGCTATTTCTAGCTC	Reverse primer 12 mer bar-code(AGCATGTCCCGT)
Illumina_rev_15	CGGTCTCGGCATTCCTGCTGAACCGCTCTTCCGATCTnnnnCCAGATATAGCAGCCTTATTTTAACTTGCTATTTCTAGCTC	Reverse primer 12 mer bar-code(CCAGATATAGCA)
Illumina_rev_16	CGGTCTCGGCATTCCTGCTGAACCGCTCTTCCGATCTnnnnGTGTCCGGATTCGCCTTATTTTAACTTGCTATTTCTAGCTC	Reverse primer 12 mer bar-code(GTGTCCGGATTC)
Illumina_rev_17	CGGTCTCGGCATTCCTGCTGAACCGCTCTTCCGATCTnnnnATCGCACAGTAAGCCTTATTTTAACTTGCTATTTCTAGCTC	Reverse primer 12 mer bar-code(ATCGCACAGTAA)
Illumina_rev_18	CGGTCTCGGCATTCCTGCTGAACCGCTCTTCCGATCTnnnnCAGCTCATCAGCGCCTTATTTTAACTTGCTATTTCTAGCTC	Reverse primer 12 mer bar-code(CAGCTCATCAGC)
Illumina_rev_19	CGGTCTCGGCATTCCTGCTGAACCGCTCTTCCGATCTnnnnGCATATGCACTGGCCTTATTTTAACTTGCTATTTCTAGCTC	Reverse primer 12 mer bar-code(GCATATGCACTG)
Illumina_rev_20	CGGTCTCGGCATTCCTGCTGAACCGCTCTTCCGATCTnnnnTGTAGGTGTGCTGCCTTATTTTAACTTGCTATTTCTAGCTC	Reverse primer 12 mer bar-code(TGTAGGTGTGCT)
Illumina_rev_21	CGGTCTCGGCATTCCTGCTGAACCGCTCTTCCGATCTnnnnACGAGACTGATTGCCTTATTTTAACTTGCTATTTCTAGCTC	Reverse primer 12 mer bar-code(ACGAGACTGATT)
DE5224	CCCTAAGAAATGAACTGGCAGC	Used in second strand synthesis reaction to make the oligo Pools of sgRNAs double stranded/Reverse primer to amplify sgRNA pool from twist
DE5231	CCTGGTTCTTGGTCTCTCAC	Forward primer to amplify sgRNA pool from twist

Name	Sequence (5’ to 3’)	Notes
DE6663	CACATATGTTACACAGGAGTCTGG ACTTGATCG	Forward primer to amplify pTox back-bone with NdeI sites in the tails to be used to clone KatG fragment
DE6664	TCACATATGGTGATGCTATAAACG CAGAAAGGCC	Reverse primer to amplify pTox back-bone with NdeI sites in the tails to be used to clone KatG fragment
DE6665	TCACATATGCAACCGTTCTAACCC GCTGG	Forward Primer to amplify KatG gene from Salmonella enterica LT2 genome with NdeI sites in the tails to be cloned into pTox backbone
DE6666	TCACATATGATCACTTTCACCCATG CCGC	Reverse Primer to amplify KatG gene from Salmonella enterica LT2 genome with NdeI sites in the tails to be cloned into pTox backbone

Table S3: Oligonucleotides used in this study.
