# Supplementary material for: A generalizable Cas9/sgRNA prediction model using machine transfer learning with small high-quality datasets
Source: Nat Commun. 2023 Sep 7;14:5514. doi: 10.1038/s41467-023-41143-7 (PMC10485023; doi:10.1038/s41467-023-41143-7)
Supplement: Supplementary file 2 — Description of Additional Supplementary Files [file 41467_2023_41143_MOESM2_ESM.pdf]

Title: Supplementary Data 1: mPool sequence IDs.
Description: List of sequences in the mPool with the number of mismatches, position of mismatches and sgRNA IDs described. The sequence 5'-CCTGGTTCTTGGTCTCTCACG'-3' was added upstream of the sgRNA and 5'-GTTTTAGAGACCGCTGCCAGTTCATTTCTTAGGG-3' was added downstream when ordering the oligo pool to allow for efficient and directional cloning.

Title: Supplementary Data 2: mPool ALDEx2 outputs.
Description: Following the enrichment experiment and Illumina sequencing of the mPool, read counts for each sgRNA in each condition were calculated for all 10 replicates and analyzed using R-package ALDEx2. The output is summarized in the table provided.

Title: Supplementary Data 3: oPool sequence IDs.
Description: List of sequences in the oPool targeting pTox plasmid with mismatch type, target strand, position in the plasmid, sgRNA ID, and notes describing the sgRNA. The sequence 5'-CCTGGTTCTTGGTCTCTCACG-3' was added upstream of the sgRNA and 5'-GTTTTAGAGACCGCTGCCAGTTCATTTCTTAGGG-3' was added downstream when ordering the oligo pool to allow for efficient and directional cloning.

Title: Supplementary Data 4: oPool TevSpCas9 and SpCas9 ALDEx2 outputs.
Description: Following the enrichment experiment and Illumina sequencing of the oPool in the SpCas9 and TevSpCas9 constructs, read counts for each sgRNA in each condition were calculated for all 10 replicates and analyzed using R-package ALDEx2. The output is summarized in the table provided.

Title: Supplementary Data 5: Predicted off-targets for sgRNAs that dropout during cloning.
Description: Off-target sites with up to six mismatches were identified in the *E. coli* genome for sgRNAs that fall out of the pool cloned into pTevSpCas9.

Title: Supplementary Data 6: Model architectures tested.
Description: Deep learning CNN, RNN, and hybrid CNN-RNN architectures tested and their respective hyperparameters.

Title: Supplementary Data 7: Model architectures performance.
Description: The 5-fold cross validation performance from the CNN, RNN, and hybrid CNN-RNN architectures tested.

Title: Supplementary Data 8: Datasets used in the construction and testing of crisprHAL.
Description: Scores, ALDEx2 effect output, and sgRNA target site sequences for the TevSpCas9, SpCas9, Guo eSpCas9, Guo SpCas9, Citrobacter TevSpCas9, and KatG fragment TevSpCas9 datasets. Original z-scores and sgRNA target site sequences for the unique Guo SpCas9 dataset.

Title: Supplementary Data 9: *Citrobacter rodentium* oPool sequence IDs.
Description: List of sequences in the oPool targeting the *Citrobacter rodentium* genome. The sequence 5'-CCTGGTTCTTGGTCTCTCACG-3' was added upstream of the sgRNA and 5'-GTTTTAGAGACCGCTGCCAGTTCATTTCTTAGGG-3' was added downstream when ordering the oligo pool to allow for efficient and directional cloning.

Title: Supplementary Data 10: *Citrobacter rodentium* oPool ALDEx2 outputs.
Description: Following the depletion experiment and Illumina sequencing of the oPool in TevSpCas9, read counts for each sgRNA in each condition were calculated for all 10 replicates and analyzed using R-package ALDEx2. The output is summarized in the table provided.

Title: Supplementary Data 11: KatG oPool sequence IDs.
Description: List of sequences in the oPool targeting the pTox+KatG plasmid. The sequence 5'-CCTGGTTCTTGGTCTCTCACG-3' was added upstream of the sgRNA and 5'-GTTTTAGAGACCGCTGCCAGTTCATTTCTTAGGG-3' was added downstream when ordering the oligo pool to allow for efficient and directional cloning.

Title: Supplementary Data 12: KatG oPool ALDEx2 outputs.
Description: Following the enrichment experiment and Illumina sequencing of the oPool in TevSpCas9, read counts for each sgRNA in each condition were calculated for all 10 replicates and analyzed using R-package ALDEx2. The output is summarized in the table provided.
